# Validity and reproducibility of the intake of *trans*-fatty acids estimated using a FFQ and characteristics of *trans*-fatty acid intake of the Japanese population: the JPHC FFQ Validation Study

**DOI:** 10.1017/S0007114522003828

**Published:** 2023-09-14

**Authors:** Kumiko Kito, Junpei Yamamoto, Ayaka Kotemori, Misako Nakadate, Koutatsu Maruyama, Saori Miyazaki, Chika Okada, Junko Ishihara, Shoichiro Tsugane, Norie Sawada

**Affiliations:** 1 Division of Cohort Research, National Cancer Center Institute for Cancer Control, 5-1-1 Tsukiji, Chuo-ku, Tokyo 104-0045, Japan; 2 Department of Food and Life Science, School of Life and Environmental Science, Azabu University, Chuo-Ku, 1-17-71 Fuchinobe, Chuo-ku, Sagamihara, Kanagawa 252-5201, Japan; 3 Department of Bioscience, Graduate School of Agriculture, Ehime University, 3-5-7 Tarumi, Matsuyama, Ehime 790-8566, Japan; 4 Department of Social Medicine, Graduate School of Medicine, Osaka University, 2-2 Yamadaoka, Suita, Osaka 565-0871, Japan; 5 National Institute of Health and Nutrition, National Institute of Biomedical Innovation, 1-23-1 Toyama, Sinjuku, Health and Nutrition, Tokyo 162-8636, Japan

**Keywords:** *Trans*-fatty acids, Industrially produced *trans*-fatty acid, Validity, FFQ, Dietary record

## Abstract

We aimed to validate a method for assessing *trans*-fatty acid (TFA) intake in the Japanese population using the FFQ developed in the 1990s from a prospective study that was based on the Japan Public Health Center-based Prospective Cohort Study. For FFQ validation, we included 565 participants (Cohort I: *n* 215, Cohort II: *n* 350) aged 40–69 years. We used a 28-d dietary record (DR) over 1 year and two FFQ administered before and after DR assessment. We calculated total TFA intake, TFA from industrial oils (i-TFA) and TFA from ruminants (r-TFA) considering a database of measurements obtained mainly from Japan. Spearman’s rank correlation coefficients (CC) were computed for validity and reproducibility. Energy adjustments were applied using two methods considering the TFA measurement: density method for TFA % of total energy and residual method for TFA g/d. The total TFA intake (% of the total energy intake) was 0·08–0·76 % (median, 0·27–0·37 %) in DR of both cohorts and was 0·00–1·13 % (median, 0·30–0·40 %) in FFQ. The i-TFA accounted for approximately 50 % of the total TFA intake in DR and approximately 40 % in FFQ. For total TFA (% of the total energy intake), CC were 0·54–0·69, and weighted *κ* coefficients were 0·88–0·92 for both cohorts. The de-attenuated CC was 0·46–0·62 for i-TFA (g/d) and 0·57–0·68 for r-TFA (g/d). Our study showed that the validity and reproducibility of TFA intake estimation using the FFQ were reasonable, suggesting its suitability among the Japanese population with low-TFA intake.

*Trans*-fatty acid (TFA) originates from two sources: naturally occurring TFA mainly found in meat and dairy products derived from ruminants (r-TFA) and industrially produced TFA (i-TFA) mainly found in margarine and vegetable oils. A recent meta-analysis showed that the intake of TFA is associated with all-cause mortality, CHD mortality and CHD occurrence^(1)^. In contrast, reductions in TFA intake lead to a decreased risk of CHD mortality^(2)^. In 2003, WHO recommended that the TFA intake of individuals should be less than 1 % of their total energy intake^(3)^. At present, WHO aims to eliminate i-TFA from food supplies by 2023^(4)^.

In the past 20 years, owing to measures such as restrictions on the use of partially hydrogenated oils in foods and mandatory labelling, TFA intake in various countries has declined^(5)^. For example, the TFA intake of high-income Western countries has decreased to approximately 1 % of total energy intake^(6)^. In Japan, TFA intake was estimated to be approximately 0·4 %^(7)^, which is lower than that worldwide, by using a 1-d dietary record (DR) from the National Health and Nutrition Examination Survey from 2003 to 2007^(7)^ and TFA concentration in foods measured by the Food Safety Commission and the Ministry of Agriculture, Forestry and Fisheries^(7)^. However, there is a lack of information regarding TFA intake before TFA reduction strategies were implemented.

A previous study reported that even low-TFA intake levels (>0·5 % of total energy) can contribute to CHD deaths^(8)^. TFA-attributable deaths were approximately 17 % in the USA, 8 % in the UK and 4 % in Japan. Therefore, the risks of disease and death in countries with low- to high-TFA intake need to be examined. Previous studies^(9–11)^ on TFA intake and disease risk have estimated exposure by using FFQ and specimens such as plasma. FFQ allow us to rank the TFA intake of the population according to their intake level and are useful in examining the association between food intake and disease risk. Therefore, FFQ are used in many epidemiological studies targeting large populations, including the Japan Public Health Center (JPHC) study. However, no studies have validated the estimated intake of TFA by FFQ in the Japanese population.

A meta-analysis also reported that CHD risk and mortality vary depending on dietary TFA sources such as industrial or ruminant^(1)^. To facilitate epidemiological studies, the validity of both TFA intake sources needs to be assessed. In the 1990s, the major i-TFA-contributing food groups in Western countries were oils and/or fats; confectionaries such as cakes, cookies and pies and chips including French fries. The major ruminant-contributing foods were dairy products, meat and meat products^(12–14)^. Because of limited data on the foods that contribute to TFA intake, it is still unclear whether the contribution of these foods to habitual TFA intake in Japan is similar to that in the Western countries.

In this study, we primarily aimed to assess the validity and reproducibility of the FFQ using JPHC 28-d DR from the late 1990s as a reference. We also examined the characteristics of TFA intake and its sources using more detailed DR because TFA intake data in Japan were insufficient in the late 1990s.

## Methods

### Study population and data collection

The JPHC study is a multipurpose cohort study that commenced in the 1990s^(15,16)^, and all participants have been followed up until now. The design and characteristics of these cohorts are described in detail elsewhere^(17–19)^. In summary, Cohort I, which launched in 1990, comprises 61 595 male and female registered residents aged 40–59 years from five public health centre areas nationwide (Ninohe, Yokote, Saku, Ishikawa and Katsushika). Cohort II, which launched in 1993, consists of 78 895 male and female registered residents aged 40–69 years from six public health centre areas (Mito, Kashiwazaki, Chuo-higashi, Kamigoto, Miyako and Suita)^(16)^. This validation study for the FFQ included a subsample from Cohorts I and II of the JPHC study. A total of 215 participants aged 40–59 years from Cohort I and 350 participants aged 40–69 years from Cohort II were included in this study. These validation studies were conducted using data from 1994 to 1996 in Cohort I (Ninohe, Yokote, Saku and Ishikawa) and data from 1996 to 1998 in Cohort II (Mito, Kashiwazaki, Chuo-higashi, Kamigoto, Miyako and Suita)^(18,19)^. Participants in this study cooperated voluntarily, and the only specific eligibility criterion was age (Cohort I: 40–59, Cohort II: 40–69). A flow diagram of the study design and participants is presented in online Supplementary Fig. 1. FFQ were administered twice, with a 1-year interval between them. DR were collected from a seven-consecutive day weighted food record that spanned across four seasons (winter, spring, summer and autumn). However, only two seasons of data were collected from the Ishikawa area as the seasonal variation was not expected to be large^(18,19)^. For validation of the FFQ, we analysed 28-d DR (14 d in Ishikawa) and two FFQ (validation; FFQ_V, reproducibility; FFQ_R). Of the 215 Cohort I participants who completed the DR and FFQ_V, 209 completed the FFQ_R. Of the 350 Cohort II participants who completed the DR and FFQ_V, 289 participants completed the FFQ_R. This study was conducted before the ethical guidelines for epidemiological research were implemented in Japan and therefore does not have ethical approval. However, informed consent was obtained from all study participants at recruitment. The protocol of the JPHC FFQ Validation Study was approved by the Institutional Review Board of the National Cancer Center, Japan.

### Database of *trans*-fatty acids

We used the TFA Food Composition database to estimate TFA intake from DR and FFQ^(20)^. Details regarding the development processes of this database have been described previously^(20)^. In summary, this database consists of the TFA concentration in foods according to twenty-two separate reports^(7,21–41)^ from Japan and the US Department of Agriculture database^(42)^. To calculate TFA intake from DR, we used the Standard Tables of Food Composition in Japan, 5th Revised and Expanded Edition (5th FCT)^(43)^ and the Fatty Acids composition table^(44)^. The 5th FCT included 1878 food items^(43)^. We allocated TFA concentrations to the food items on the FCT list. Of the 1878 foods, 594 (32 %) were identified as TFA-containing foods, 877 foods were treated as non-TFA-containing foods (foods with less than 1 % of fat per 100 g of foods) and 407 foods were treated as having missing values because these foods have no information regarding TFA concentration.

### Estimation of *trans*-fatty acids using the dietary records

Study participants recorded menu names of foods and beverages and weights of foods in their DR. The dietitian checked the DR and coded each food item using the 5th FCT^(43)^. TFA intake was calculated by multiplying the weight of each food by its TFA concentration from a database of TFA-containing foods. These values were summed for each day and divided by the total number of DR days to calculate the mean daily value. Energy intake was also calculated using the 5th FCT^(43)^.

### Estimation of *trans*-fatty acids using the FFQ

The FFQ assesses habitual dietary intake over the past year^(18,19)^. In brief, the FFQ was developed from a weighted 3-d DR of 383 men and women in a pilot study in Cohort I from 1989 to 1991^(45)^. The food items in the FFQ were chosen with the aim of covering 80 % of the total population’s intake of energy including each nutrient. The internal validation study was conducted in Cohort I from 1994 to 1996^(18)^. The external validation study was conducted in Cohort II from 1996 to 1998^(19)^.

Of all 147 food items included in FFQ, TFA was found in the following thirty-nine foods (27 %): beef dishes (steak, grilled beef and stewed beef), pork dishes (stir-fried pork, deep-fried pork, stewed pork in Western style, stewed pork in Japanese style, pork in soup and pork liver), chicken dishes (grilled chicken and chicken liver), processed meat products (ham, sausage or wiener sausage, bacon and luncheon meat), milk, eggs, cheese, yogurt, cod, sea bream, cod roe, eel, squid, fish sausage, bread, cakes, biscuits and cookies, chocolates, peanuts, fried tofu, butter, margarine, salad dressing, mayonnaise, soup, milk for tea, milk for coffee and vegetable oil. The TFA intake was estimated by multiplying the TFA concentration per 100 g of food by the frequency and portion size of each food/beverage for each participant.

### *Trans*-fatty acid from an industrial source

Referring to the Food Safety Commission of the Cabinet Office report in Japan^(7)^, the following foods were selected as i-TFA-containing foods: processed oils and fats (margarine and animal fats), foodstuffs containing processed oils and fats (bread, sweetbreads, instant noodles, cakes and pastries, cookies and other confectioneries), vegetable oils (vegetable oils and mayonnaise) and foodstuffs containing vegetable oil (fried foods). The number of foods containing i-TFA was 91 of 594 in the DR and 10 of 39 in the FFQ.

### *Trans*-fatty acid from a ruminant source

Referring to the Food Safety Commission of the Cabinet Office report in Japan^(7)^, the following foods were selected as r-TFA: milk, cheese, fermented milk/lactic acid drinks, other dairy products, butter, beef and beef offal. The number of foods containing r-TFA was 143 of 594 in the DR and 10 of 39 in the FFQ.

### Statistical analysis

Mean TFA intake was estimated by cohort and sex. Spearman’s rank correlation coefficients (CC) between DR and FFQ were calculated for TFA (%) of the total energy and TFA intake (g). We used the residual method for energy adjustment for TFA intake (g). Furthermore, we calculated de-attenuated CC to correct for the attenuating effect of random within-person error. The equation used was as follows: de-attenuated CC = observed energy-adjusted CC × square root (1 + *λ*x/*n*), where *λ*x is the ratio of within- and between-person variance of DR and *n* is the number of DR per person (28 d). In a cross-classification analysis, energy-adjusted TFA intake from both DR and the FFQ was grouped into quintiles and calculated as the proportion (%) of participants among the same, adjacent and opposite categories according to the quintiles of DR and the FFQ. In addition, a weighted *κ* coefficient was calculated to confirm the degree of agreement for the ranking of TFA between DR and the FFQ. This analysis was performed using SAS 9.4 (SAS Institute Inc.).

## Results

### Characteristics of the participants

The basic characteristics of the participants have been described in detail elsewhere^(18,19)^. The participants’ energy and fat intake characteristics are summarised in Table 1. The mean total fat was 23·2 % of the total energy for men and 26·4 % of the total energy for women in Cohort I, and 22·0 % of the total energy for men and 24·7 % of the total energy for women in Cohort II.

**Table 1.** Energy intake and fat intake according to DR and the FFQ (Mean values and standard deviations; median values)

	DR					FFQ				
	Mean	sd	Median	25th	75th	Mean	sd	Median	25th	75th
Cohort I										
Men (*n* 102)										
Energy (kJ)	10016	1824	9954	8786	11301	9690	2774	9464	7904	10996
Total fat (% total energy)	23·2	4·5	22·4	20·2	25·9	23·6	6·5	23·5	19·0	27·4
Total fat (g)	60·5	11·0	60·6	52·7	68·1	61·5	26·4	58·0	43·1	72·6
SFA (g)	16·6	3·9	16·4	13·7	19·1	18·4	9·0	17·0	12·0	21·8
MUFA (g)	21·1	4·2	20·8	17·8	24·0	21·2	9·4	20·6	14·7	25·3
PUFA (g)	14·5	2·7	14·4	12·6	16·3	14·2	5·9	13·5	9·9	17·1
Women (*n* 113)										
Energy (kJ)	7778	1347	7724	6799	8535	8251	3326	7736	6406	9209
Total fat (% total energy)	26·4	4·3	25·8	23·6	28·7	27·1	5·5	26·5	23·0	30·4
Total fat (g)	54·0	10·5	53·6	45·8	59·5	60·7	33·2	53·1	43·6	69·1
SFA (g)	15·2	3·7	14·6	13·1	17·3	18·0	9·1	15·8	12·8	20·6
MUFA (g)	18·6	4·1	18·2	16·2	20·6	20·7	11·9	17·6	14·1	23·5
PUFA (g)	12·8	2·5	12·6	11·2	14·0	14·2	8·6	12·1	10·0	15·6
Cohort II										
Men (*n* 174)										
Energy (kJ)	9535	1490	9506	8535	10418	9209	2720	8899	7222	10602
Total fat (% total energy)	22·0	4·1	22·1	19·0	24·8	24·2	6·0	24·6	19·9	28·5
Total fat (g)	55·3	11·9	55·0	47·1	63·2	60·0	25·8	55·2	42·5	74·2
SFA (g)	14·9	3·8	14·7	12·0	17·5	18·5	9·0	16·1	12·6	23·0
MUFA (g)	19·5	4·7	19·3	16·2	22·4	20·6	9·3	19·1	14·2	25·3
PUFA (g)	13·3	2·9	13·2	11·2	15·2	13·3	5·5	12·7	9·4	16·2
Women (*n* 176)										
Energy (kJ)	7406	1079	7318	6703	8042	7724	2736	7217	6280	8598
Total fat (% total energy)	24·7	3·9	24·8	21·9	27·6	27·6	5·9	27·7	24·2	31·2
Total fat (g)	48·6	10·0	48·4	41·6	54·9	58·6	29·6	52·2	41·0	68·4
SFA (g)	13·5	3·5	13·5	11·0	15·6	18·2	10·1	16·2	11·7	21·9
MUFA (g)	16·7	3·7	16·7	14·1	19·3	19·8	10·8	17·3	13·5	23·1
PUFA (g)	11·4	2·3	11·4	9·8	13·1	13·0	6·3	11·8	9·7	14·7

DR, dietary record

### Intake of *trans*-fatty acids

The estimated intake of TFA using DR and FFQ is presented in Table 2. In Cohort I, the men’s TFA intake in the DR was 0·10–0·64 % of the total energy, with a median (IQR, interquartile range) of 0·27 % (0·23 %, 0·37 %) of the total energy. The women’s TFA intake was 0·11–0·76 % of the total energy, with a median (IQR) of 0·35 % (0·29 %, 0·43 %) of the total energy. In Cohort II, the men’s TFA intake in the DR was 0·08–0·70 % of the total energy, with a median (IQR) of 0·29 % (0·23 %, 0·38 %) of the total energy. The women’s TFA intake was 0·13–0·75 % of the total energy, with a median (IQR) of 0·37 % (0·29 %, 0·45 %) of the total energy. None of the participants had TFA acid intake of more than 1 % of the total energy intake in the DR regardless of cohort. Compared with that in DR, the total TFA intake in the FFQ (% of the total energy) was overestimated by approximately 4 % in Cohort I and by approximately 15 % in Cohort II. The intake from industrial sources accounted for approximately 50 % of the total TFA intake regardless of the cohort or sex. The intake of TFA according to age group is summarised in online Supplementary Table S1.

**Table 2.** Intake of *trans*-fatty acid estimated using DR and the FFQ (Mean values and standard deviations; median values)

	DR							FFQ							Percentage differences (%)*
	Mean	sd	Minimum	Median	25th	75th	Maximum	Mean	sd	Minimum	Median	25th	75th	Maximum	
Cohort I															
Total *trans*-fatty acids (% total energy)															
Men (*n* 102)	0·30	0·11	0·10	0·27	0·23	0·37	0·64	0·32	0·15	0·09	0·30	0·21	0·39	0·79	4
Women (*n* 113)	0·38	0·12	0·11	0·35	0·29	0·43	0·76	0·39	0·15	0·16	0·36	0·28	0·48	0·87	4
Total *trans*-fatty acids (g/d)															
Men (*n* 102)	0·80	0·28	0·28	0·76	0·60	0·96	1·66	0·81	0·46	0·16	0·67	0·48	1·05	2·52	1
Women (*n* 113)	0·79	0·25	0·23	0·77	0·58	0·95	1·47	0·87	0·51	0·22	0·74	0·53	1·08	3·21	10
Industrial *trans*-fatty acids (g/d)															
Men (*n* 102)	0·39	0·17	0·13	0·36	0·27	0·47	0·99	0·30	0·24	0·05	0·23	0·15	0·36	1·43	−24
Women (*n* 113)	0·43	0·18	0·13	0·40	0·29	0·52	1·07	0·38	0·31	0·06	0·27	0·18	0·49	1·69	−11
Ruminant *trans*-fatty acids (g/d)															
Men (*n* 102)	0·29	0·16	0·02	0·24	0·19	0·37	0·75	0·39	0·30	0·00	0·31	0·20	0·51	2·32	37
Women (*n* 113)	0·25	0·12	0·03	0·23	0·17	0·33	0·63	0·37	0·24	0·04	0·31	0·19	0·45	1·21	47
Cohort II															
Total *trans*-fatty acids (% total energy)															
Men (*n* 174)	0·31	0·11	0·08	0·29	0·23	0·38	0·70	0·36	0·15	0·09	0·34	0·25	0·46	0·82	15
Women (*n* 176)	0·37	0·12	0·13	0·37	0·29	0·45	0·75	0·43	0·16	0·00	0·40	0·32	0·53	1·13	15
Total *trans*-fatty acids (g/d)															
Men (*n* 174)	0·79	0·29	0·19	0·72	0·58	0·99	1·80	0·87	0·44	0·19	0·77	0·54	1·13	2·31	10
Women (*n* 176)	0·76	0·27	0·22	0·73	0·58	0·90	1·81	0·91	0·50	0·00	0·83	0·56	1·16	4·35	19
Industrial *trans*-fatty acids (g/d)															
Men (*n* 174)	0·41	0·19	0·10	0·38	0·28	0·49	1·18	0·33	0·24	0·04	0·26	0·17	0·39	1·33	−21
Women (*n* 176)	0·42	0·20	0·05	0·38	0·29	0·51	1·18	0·40	0·28	0·00	0·31	0·21	0·54	2·09	−6
Ruminant *trans*-fatty acids (g/d)															
Men (*n* 174)	0·24	0·13	0·02	0·22	0·14	0·30	0·02	0·41	0·29	0·01	0·32	0·22	0·53	2·20	71
Women (*n* 176)	0·23	0·12	0·02	0·22	0·14	0·30	0·02	0·39	0·27	0·00	0·32	0·23	0·50	2·12	69

DR, dietary record.

* Percentage differences (%) were calculated using the following formula: (‘mean FFQ’ − ‘mean DR’)/‘mean DR’ × 100.

### Validity and reproducibility of the FFQ

Table 3 shows the validity, cross-classification analysis and reproducibility of the FFQ. In Cohorts I and II, the CC (% of total energy) was 0·54–0·69, energy-adjusted CC (g/d) was 0·56–0·67 and de-attenuated CC (g/d) was 0·60–0·73. For cross-classification analysis of the total TFA intake (g/d) assessed in Cohorts I and II using the FFQ and DR, the following results were obtained for categories: ‘same’, 34–41 % of participants; ‘same and adjacent’, 71–79 % and ‘extreme’, 0–1 %. The weighted *κ* coefficient for total TFA intake (% of the total energy) was approximately 0·9 in both cohorts. For reproducibility, the energy-adjusted CC (% of the total energy) between two FFQ was 0·64–0·75.

**Table 3.** Correlation between DR and FFQ_V and between two repeated FFQ and cross-classification analysis

	DR *v*. FFQ_V			Cross-classification (%)†			Weighted *κ* coefficient	FFQ_V *v*. FFQ_R	
	Validity*							Reproducibility‡	
	Crude	Energy-adjusted	De-attenuated§	Same category	Same and adjacent category	Extreme category		Crude	Energy-adjusted
Cohort I									
Total *trans*-fatty acids (% total energy)									
Men (*n* 102)	0·67	–	–	47	78	0	0·91	0·70	–
Women (*n* 113)	0·69	–	–	42	81	0	0·92	0·71	–
Total *trans*-fatty acids (g/d)									
Men (*n* 102)	0·55	0·67	0·73	39	79	0	0·90	0·61	0·70
Women (*n* 113)	0·45	0·66	0·73	41	71	0	0·91	0·66	0·71
Industrial *trans*-fatty acids (g/d)									
Men (*n* 102)	0·44	0·56	0·62	25	75	0	0·89	0·62	0·76
Women (*n* 113)	0·30	0·41	0·46	22	65	3	0·85	0·65	0·52
Ruminant *trans*-fatty acids (g/d)									
Men (*n* 102)	0·59	0·60	0·68	41	76	0	0·89	0·61	0·59
Women (*n* 113)	0·61	0·60	0·68	35	77	1	0·89	0·63	0·64
Cohort II									
Total *trans*-fatty acids (% total energy)									
Men (*n* 174)	0·65	–	–	45	80	1	0·91	0·75	–
Women (*n* 176)	0·54	–	–	35	75	1	0·88	0·64	–
Total *trans*-fatty acids (g/d)									
Men (*n* 174)	0·47	0·61	0·67	40	79	1	0·90	0·66	0·73
Women (*n* 176)	0·41	0·56	0·60	34	74	1	0·88	0·60	0·68
Industrial *trans*-fatty acids (g/d)									
Men (*n* 174)	0·41	0·46	0·50	33	71	2	0·86	0·62	0·62
Women (*n* 176)	0·39	0·48	0·53	31	74	2	0·87	0·58	0·65
Ruminant *trans*-fatty acids (g/d)									
Men (*n* 174)	0·49	0·50	0·57	33	75	2	0·87	0·63	0·61
Women (*n* 176)	0·50	0·54	0·60	36	76	2	0·88	0·67	0·64

DR, dietary record; FFQ_V, FFQ for validity study; FFQ_R, FFQ for reproducibility.

* Spearman’s correlation between DR and FFQ_V.

† Percentage shows the degree of agreement in the cross-classification according to the quintile of DR and the FFQ_V.

‡ Spearman’s correlation of the two repeated FFQ.

§ De-attenuated CC = energy-adjusted CC × 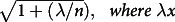 is the ratio of within- to between-individual variance and *n* is the number of DR.

For i-TFA and r-TFA, in Cohorts I and II, the de-attenuated CC for i-TFA (g/d) was 0·46–0·62 and for r-TFA (g/d) was 0·57–0·68. For reproducibility between two FFQ, the energy-adjusted CC for i-TFA (g/d) was 0·52–0·76 and for r-TFA (g/d) was 0·59–0·64.

### Main source of *trans*-fatty acids

The primary food groups that contributed to TFA intake were fats and/or oil, meats, milk and dairy products and confectioneries (Table 4). Of these groups, the primary sources of i-TFA were vegetable oil, margarine, processed roux, dressing, cake and/or pastry and biscuits. The primary sources of r-TFA were beef and milk. These eight foods accounted for approximately 70 % of the total TFA intake regardless of sex.

**Table 4.** Food groups (%) contributing to total *trans*-fatty acids intake estimated from DR

	Proportion (%)	Top 5 foods (%)									
		1		2		3		4		5	
Men (*n* 276)											
Fats and oils	30·4	Vegetable fats and oils	22·3	Margarines	5·6	Butters	2·2	Animal fats	0·2		
Meats	22·4	Beefs	13·9	Pork	7·4	Chicken	1·0	Sheep	0·1		
Milk and dairy products	15·6	Milk	10·2	Cheese	2·5	Ice cream	0·8	Yogurt	0·7	Coffee whitener	0·5
Seasoning and spices	9·5	Roux	5·9	Dressings	3·5	Spices	0·05				
Confectioneries	7·8	Cakes, buns and pastries	4·3	Biscuits	2·5	Traditional fresh and semi-dry confectioneries	0·4	Chocolates	0·2	Snacks	0·2
Fishes and shellfishes	6·2	Japanese eel	4·0	Fish paste products	1·0	Fish roe	0·7	Red sea bream	0·4	Oysters	0·1
Cereals	3·5	Breads	2·4	Noodles dried by flying	0·4	Wheat flour	0·3	Bread crumbs	0·3	Steamed Chinese noodles	0·05
Eggs	2·1	Eggs	2·1								
Pulses	1·5	Tofu (fried)	1·5								
Potatoes and starches	0·9	Potatoes	0·9								
Women (*n* 289)											
Fats and oils	28·0	Vegetable fats and oils	18·9	Margarines	6·7	Butters	2·3	Animal fats	0·2		
Milk and dairy products	19·1	Milk	12·3	Cheese	2·4	Ice cream	1·3	Yogurt	1·1	Creams	0·8
Meats	16·7	Beef	9·7	Pork	6·0	Chicken	0·9	Sheep	0·1	Duck	0·01
Confectioneries	14·6	Cakes, buns and pastries	8·0	Biscuits	5·0	Traditional fresh and semi-dry confectioneries	0·6	Chocolates	0·3	Traditional dry confectioneries	0·2
Seasoning and spices	9·1	Roux	5·2	Dressings	3·9	Spices	0·04				
Fishes and shellfishes	4·8	Japanese eel	2·8	Fish paste products	1·0	Fish roe	0·5	Red sea bream	0·2	Oysters	0·04
Cereals	4·0	Breads	3·1	Wheat flour	0·3	Bread crumbs	0·3	Noodles dried by flying	0·3	Steamed Chinese noodles	0·04
Eggs	1·7	Eggs	1·7								
Pulses	1·4	Tofu (fried)	1·4								
Potatoes and starches	0·7	Potatoes	0·7								

DR, dietary record.

## Discussion

This study examined the validity and reproducibility of estimated TFA intake using an FFQ. The results showed moderate correlations between DR and the FFQ and between two FFQ and showed high weighted *κ* coefficients. The total TFA intake (% of the total energy intake) ranged from 0·08 to 0·76 % in DR of both cohorts and ranged from 0·00 to 1·13 % in FFQ. The main contributing food groups were fats and oils, meat, milk and dairy products, seasoning and spices and confectioneries.

Our results are similar to the CC in several previous validation studies on total TFA between the FFQ and DR. For energy-adjusted CC, the Dutch cohorts of the European Prospective Investigation into Cancer and Nutrition cohort (EPIC) reported 0·53 for men and 0·49 for women^(46)^, the Adventist Health Study-2 in the USA and Canada reported 0·35 for both Blacks and Whites^(47)^ and a South Korean study among healthy medical students and nurses reported 0·45^(48)^. The percentage difference in the estimation of total TFA intake (g/d) between the FFQ and DR in the current study was approximately 20 %, and the distribution was similar. Thus, we consider that the assessment using an FFQ is relatively accurate. Owing to a lack of similar studies, it is difficult to compare the validity of i-TFA in this study with that in other studies; however, moderate correlations and high-weighted *κ* coefficients were obtained in this study. Overall, it is likely that this FFQ is applicable to rank TFA intake among the population in epidemiological studies, especially in JPHC studies.

The mean TFA intake estimated using the DR in the late 1990s was approximately 0·3–0·4 % of total energy, and no participant had TFA intake of more than 1 % of total energy, in accordance with WHO recommendations. This figure is lower than that observed in the Western countries within the same period (1–2 % of total energy) and the latest data (0·3–4·2 % of total energy)^(6)^. Meanwhile, compared with a previous Japanese study within the same period (0·3–0·5 % of total energy)^(49)^ and the data between 2002 and 2012 (0·14–0·90 % of total energy)^(49–52)^, the total TFA intake was almost the same. Although the proportion of people with TFA intake of 1 % or more of total energy in each country is still unclear, according to the review^(6)^, in Western countries, the 95th percentile of TFA intake declined. For example, in France, TFA comprised 2·0 % of total energy in 1998–1999 and 1·2 % of total energy in 2006–2007^(6)^. In Japan, the proportion of those who exceeded 1 % of total energy intake was 1·8–24·4 % between 2003 and 2012^(49–52)^. Owing to the limited availability of data, it is essential to understand TFA intake and the number and/or the proportion of the population exceeding 1 % of total energy intake to promote the WHO’s recommendation for TFA intake.

The range of distribution in TFA intake by DR was wide in both cohorts (approximately 0·1–0·8 % of total energy). Total TFA intake among women was higher than that among men, and total TFA intake among those in their 40s was higher than that among those in their 60s. These characteristics were consistent with those in a previous study in Japan^(50)^ that used 16-d DR from 2000 to 2003: distribution (0·2–1·9 % of total energy); sex-specific difference (women in their 40s: 0·9 % of total energy *v*. men in their 40s: 0·7 % of total energy) and age differences (women in their 30s: 1·0 % of total energy *v*. women in their 60s: 0·6 % of total energy). However, our estimated TFA intake may be underestimated for the following two reasons. First, the previous study^(50)^ reported lower TFA intake in rural areas than in urban areas. Most of our participants lived in rural areas. Second, the reports used in developing the TFA database included measurements taken during the period following this study when TFA levels in foods had been reduced voluntarily by food manufacturers^(53)^. Therefore, the i-TFA intake of those who consume foods high in i-TFA may be underestimated. Hence, we need to consider both the characteristics of the population and the background of the database: (1) when estimating the intake and comparing the results to those of other studies and (2) when estimating the disease risks.

Regarding intake of i-TFA, the recent EPIC study on i-TFA intake and breast cancer risk reported a relatively high risk from the second quintile and higher (reference group: <0·54 g/d)^(54)^. However, the risk in the group with lower TFA intake than the group at risk in the EPIC study is unknown. The mean i-TFA in this study was approximately 0·39–0·43 (sd 0·17–0·20) g/d, which is lower than the aforementioned reference group. Accordingly, there may be added evidence on the association between i-TFA and disease risk in the population with relatively low intake using the validated FFQ.

The primary TFA-containing foods derived from industrial oils were vegetable oil, margarine, processed roux, dressing, cake and/or pastry and biscuits, and those derived from ruminants were beef and milk. Primary foods in the FFQ were also similar to those in the DR (data not shown). These sources are similar to those reported in several previous studies in Europe^(12)^, the USA^(13)^ and China^(55)^. This study did not identify foods that remarkably contribute to TFA intake. However, even with foods that contribute equally to TFA intake, their ranking may vary depending on the country’s dietary background^(12,13,55)^. For example, among the Belgians and Dutch, French fries and chips contribute more significantly to their TFA intake than that observed among other Western Europeans^(12)^. In the Chinese population, vegetable oil accounts for 70 % of TFA intake^(55)^. Hence, studies aimed at reducing TFA, and policies intending to direct the same, should consider each country’s dietary background.

This study has several strengths. First, we used long-term DR (28 d a year, 7 d for each season), resulting in less measurement error than that observed with short-term DR^(56)^. Second, the current data were collected in the 1990s before various measures were taken to reduce TFA intake. Information on TFA intake in the Japanese population within this period was limited; thus, these data allow comparisons with other periods.

There are some limitations to our study. The first is a limitation attributed to the TFA database. Although our dietary surveys were conducted in the 1990s, the database of TFA consists of measurements available from reports in the 1990s–2010s. Since the 2000s, efforts to reduce TFA intake, such as the changing of recipes in food processing, have been reported in Japan^(57)^. For example, some food-producing manufacturers have replaced partially hydrogenated oils with palm oil^(58)^. Therefore, estimated intake of TFA and TFA-contributing foods, especially i-TFA, may be underestimated. The TFA database also covered only approximately 32 % of the items in DR in the 5th FCT and 27 % of the items in the FFQ; thus, we could not calculate TFA intake from unlisted foods that may contain TFA. In addition, TFA concentration in foods in the same category varies from product to product. We used mean TFA concentration to reduce the effect of such variations. Second, the food lists of this FFQ include the foods that highly contribute to the targeted population’s primary nutritional intake (not including TFA intake); therefore, we could not consider TFA intake from uncommonly eaten foods. Third, we could not consider the intake of TFA among participants with a specific dietary therapy; such therapies may restrict foods that are rich in TFA. Finally, we could not examine the validity of the FFQ using biomarkers such as adipose tissue and plasma. For example, plasma elaidic acid concentration, which is a C18:1 isomer of TFA, is a characteristic biomarker for i-TFA intake. We believe that we can support the accuracy of TFA estimations from the FFQ by examining the relationship between these concentrations in plasma and food intake.

Regarding the validity and reproducibility of the FFQ, total intake of TFA and industrial TFA showed moderate correlations. Therefore, this FFQ is applicable in ranking the TFA intake of the population in epidemiological studies. In addition, the percentage differences in the estimation of total TFA intake (g/d) between the FFQ and DR are approximately 20 %. Therefore, we consider that the TFA intake estimated using the FFQ is relatively accurate. Although total TFA intake and i-TFA were estimated to be low in the current study, there is a need to examine the association between TFA intake and disease risks in the future because low i-TFA intake may still be a risk factor for disease^(54)^.

## Supplementary material

For supplementary material accompanying this paper visit https://doi.org/10.1017/S0007114522003828. click here to view supplementary material
