# Supplementary material for: Validity and reproducibility of the intake of trans-fatty acids estimated using a FFQ and characteristics of trans-fatty acid intake of the Japanese population: the JPHC FFQ Validation Study
Source: Br J Nutr. 2022 Dec 1;130(5):895–903. doi: 10.1017/S0007114522003828 (PMC10404479; doi:10.1017/S0007114522003828)
Supplement: Supplementary file 1 [file S0007114522003828sup001.pptx]

## Slide 1
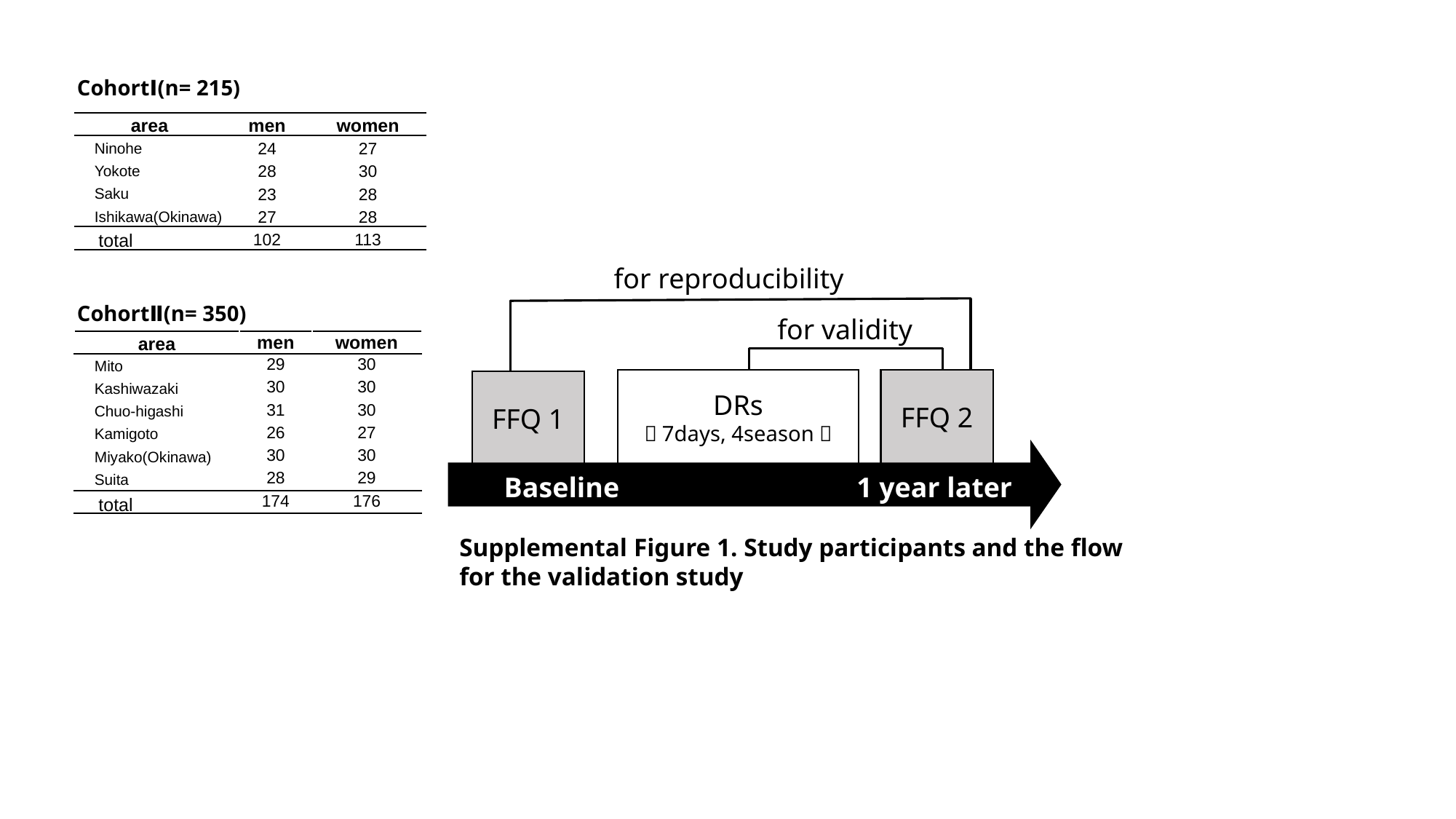

CohortⅠ(n= 215)
| area | men | women |
| --- | --- | --- |
| Ninohe | 24 | 27 |
| Yokote | 28 | 30 |
| Saku | 23 | 28 |
| Ishikawa(Okinawa) | 27 | 28 |
| total | 102 | 113 |
for reproducibility
for validity
DRs
（7days, 4season）
FFQ 2
FFQ 1
Baseline
1 year later
CohortⅡ(n= 350)
| area | men | women |
| --- | --- | --- |
| Mito | 29 | 30 |
| Kashiwazaki | 30 | 30 |
| Chuo-higashi | 31 | 30 |
| Kamigoto | 26 | 27 |
| Miyako(Okinawa) | 30 | 30 |
| Suita | 28 | 29 |
| total | 174 | 176 |
Supplemental Figure 1. Study participants and the flow for the validation study
